# Cellulosic Ethanol Production Through Cocultivation of *Thermoanaerobacter brockii* subsp. *finnii* DSM 3389 and *Acetivibrio thermocellus* JYT01

**DOI:** 10.3390/microorganisms14102164

**Published:** 2026-09-25

**Authors:** Zili Qiu, Chenggang Xu, Zhenxing Ren

**Affiliations:** 1 Institute of Applied Chemistry, Shanxi University, Taiyuan 030006, China; 2 College of Animal Science and Technology & College of Veterinary Medicine of Zhejiang A&F University, Key Laboratory of Applied Technology on Green-Eco-Healthy Animal Husbandry of Zhejiang Province, Zhejiang Provincial Engineering Research Center for Animal Health Diagnostics & Advanced Technology, Zhejiang International Science and Technology Cooperation Base for Veterinary Medicine and Health Management, China-Australia Joint Laboratory for Animal Health Big Data Analytics, Hangzhou 311300, China

**Keywords:** cellulosic ethanol, consolidated bioprocessing, defined microbial consortium, thermophilic fermentation, *Thermoanaerobacter brockii*, *Acetivibrio thermocellus*, inhibitor tolerance

## Abstract

Consolidated bioprocessing integrates enzyme production, cellulose hydrolysis, and fermentation in a single reactor; however, few naturally occurring microorganisms combine efficient cellulose degradation, broad sugar utilization, inhibitor tolerance, and ethanol production. In this study, seven thermophilic ethanologenic strains were compared for sugar utilization, ethanol production, and tolerance to furfural, *p*-hydroxybenzoic acid, ethanol, formate, acetate, and lactate. Four strains showed sugar-to-ethanol conversion ratios above 32% in a glucose and xylose mixture. Among them, *Thermoanaerobacter brockii* subsp. *finnii* DSM 3389 showed the highest conversion ratio of 35.7% and retained growth and ethanol production in the presence of 20 mM furfural or 20 mM *p*-hydroxybenzoic acid. DSM 3389 also grew in the presence of approximately 2.5% (*v*/*v*) exogenous ethanol and in media containing 100 mM sodium formate, sodium acetate, or sodium lactate. The strain was subsequently cocultivated with *Acetivibrio thermocellus* JYT01 in a single preliminary 2 L pH-controlled fermentation containing 40 g/L Avicel. In this run, the ethanol concentration at 72 h was 10.86 g/L, corresponding to a mass-based cellulose-to-ethanol conversion ratio of 27.2% based on the initial Avicel concentration. These results demonstrate ethanol production by the DSM 3389–JYT01 coculture and support further evaluation of DSM 3389 as an ethanologenic partner for thermophilic cellulose conversion.

## 1. Introduction

Efficient conversion of lignocellulosic biomass into liquid transport fuels is important for developing sustainable alternatives to petroleum-based fuels. Cellulosic ethanol production generally begins with pretreatment of lignocellulose, followed by cellulose and hemicellulose hydrolysis and fermentation of the released pentoses and hexoses [1,2,3]. Consolidated bioprocessing (CBP) integrates enzyme production, polysaccharide hydrolysis, and microbial fermentation in a single reactor [4,5,6]. This process can reduce the requirement for externally produced cellulases and simplify the overall process configuration. Nevertheless, insufficient ethanol yield, titer, and productivity at industrially relevant substrate loadings remain important limitations [1,2].

Thermophilic anaerobic microorganisms are attractive candidates for CBP because several species possess cellulolytic or ethanologenic properties and can operate at elevated temperatures [6,7,8]. *Acetivibrio thermocellus* is one of the most extensively studied cellulolytic thermophiles. Its cellulosome enables rapid degradation of crystalline cellulose, but the organism has a restricted range of fermentable soluble sugars and produces organic acids and other metabolites in addition to ethanol [9,10,11]. Metabolic and evolutionary engineering has improved ethanol production and tolerance in *A. thermocellus*, although redox balance, product distribution, and ethanol toxicity remain important constraints [12,13,14].

Cocultivation provides an alternative strategy in which a cellulolytic microorganism hydrolyses cellulose and a second microorganism converts the released sugars into ethanol. Previous studies have shown that cocultivation of *A. thermocellus* with thermophilic saccharolytic bacteria can improve soluble-sugar utilization and ethanol production under certain cultivation conditions [15,16,17]. More recent studies have emphasized the importance of cross-feeding, complementary enzymatic activities, feedstock composition, and population interactions in thermophilic cocultures [18,19,20]. However, the performance of such a coculture depends strongly on the physiological properties of the ethanologenic partner. In addition to efficient conversion of both glucose and xylose, a suitable partner should maintain growth and ethanol production in the presence of pretreatment-derived furans and phenolic compounds, as well as ethanol and organic acids generated during fermentation [21,22,23]. Because the composition and concentrations of these inhibitors vary with feedstock and pretreatment conditions, comparative evaluation under common cultivation conditions is important for strain selection [24,25,26].

In search of a suitable ethanologenic partner for *A. thermocellus* JYT01 in a defined thermophilic coculture for CBP [27], we hypothesized that a thermophilic ethanologen combining efficient glucose and xylose conversion with tolerance to pretreatment-derived inhibitors and fermentation products would be a suitable coculture partner for JYT01. To test this hypothesis, seven thermophilic ethanologenic strains were compared under common cultivation conditions for sugar utilization, ethanol production, and tolerance to furfural, *p*-hydroxybenzoic acid, ethanol, formate, acetate, and lactate. A selected candidate was subsequently evaluated with JYT01 in a preliminary pH-controlled coculture fermentation on microcrystalline cellulose. This study provides a comparative strain-level assessment for selecting an ethanologenic partner and a preliminary evaluation of the resulting defined thermophilic coculture.

## 2. Materials and Methods

### 2.1. Bacterial Strains and Culture Media

The thermophilic ethanologenic strains evaluated in this study included *Thermoanaerobacter* sp. X514 [28], *Thermoanaerobacterium xylanolyticum* DSM 7097 [29], *Thermoanaerobacter brockii* subsp. *finnii* DSM 3389 [30], *Thermoanaerobacter italicus* DSM 9252 [29], *Caldanaerobius polysaccharolyticus* DSM 13641 [29], *Thermoanaerobacter thermocopriae* DSM 11426 [31], and *Thermoanaerobacter wiegelii* DSM 10319 [30]. These strains were purchased from the German Collection of Microorganisms and Cell Cultures (DSMZ).

*Acetivibrio thermocellus* JYT01 was used as the cellulose-degrading partner. JYT01 is a laboratory derivative of strain LQR1 and was previously reported to tolerate up to 3% (*w*/*v*) ethanol [27]. All strains were grown anaerobically in GS-2 medium. For bottle cultures, GS-2 medium was dispensed into serum bottles, flushed with nitrogen to remove oxygen, and sealed with butyl rubber stoppers and aluminum crimps before sterilization. L-Cysteine and resazurin in the GS-2 medium served as the reducing agent and redox indicator, respectively [27,32]. Only bottles in which the resazurin remained colorless were inoculated. Glucose, xylose, or microcrystalline cellulose was used as the carbon source as specified below. Avicel PH-101, with an approximate particle size of 50 μm, was obtained from Sigma-Aldrich. Other chemicals were of analytical grade.

### 2.2. Growth Conditions for Thermophilic Ethanologenic Strains

All sugar-utilization and inhibitor-tolerance experiments were performed at 60 °C in 70 mL serum bottles containing 30 mL GS-2 medium. Activated cultures were inoculated to an initial optical density at 600 nm, OD_600_, of 0.01. For single-sugar experiments, either 20 g/L glucose or 20 g/L xylose was used as the sole carbon source. Mixed-sugar cultures contained 10 g/L glucose and 10 g/L xylose. Samples were collected every 8 h for measurements of OD_600_, residual sugar, and ethanol.

Four strains selected from the sugar-utilization experiments, *T. brockii* subsp. *finnii* DSM 3389, *T. xylanolyticum* DSM 7097, *T. italicus* DSM 9252, and *T. thermocopriae* DSM 11426, were evaluated for inhibitor tolerance in GS-2 medium containing 10 g/L glucose. Sodium formate, sodium acetate, and sodium lactate were tested separately at concentrations ranging from 0 to 100 mM. Exogenous ethanol was tested at concentrations ranging from 0 to 3.0% (*v*/*v*). Furfural was tested at concentrations ranging from 0 to 40 mM, and *p*-hydroxybenzoic acid was tested at concentrations ranging from 0 to 50 mM.

The initial pH of the GS-2 medium was 7.4. Microbial growth was evaluated by measuring OD_600_ with a GeneQuant 1300 spectrophotometer (Biochrom Ltd., Cambridge, UK). Growth curves in the ethanol-tolerance experiment were fitted using the exponential growth equation *Y = ae^bX^*. Doubling time was calculated as ln(2)/*b*.

### 2.3. Mixed Fermentation of DSM 3389 and JYT01

A single preliminary coculture fermentation was conducted. Seed cultures of *T. brockii* subsp. *finnii* DSM 3389 and *A. thermocellus* JYT01 were prepared separately in GS-2 medium. A 5 L bioreactor containing 2 L GS-2 medium and 40 g/L Avicel PH-101 was flushed with nitrogen before inoculation to remove dissolved oxygen and establish anaerobic conditions. The reactor was inoculated with 100 mL of each seed culture, giving an initial inoculum ratio of 1:1 (*v*/*v*).

Fermentation was performed at 60 °C with stirring at 100 rpm. The pH was maintained at 7.0 by automatic addition of 1 M NaOH or 1 M HCl. Samples of 5 mL were collected every 12 h for substrate and metabolite analyses.

### 2.4. Chemical Analysis

Total reducing sugars were measured using a modified 3,5-dinitrosalicylic acid method as described previously [27,33]. Glucose was measured using the K-GLUC assay kit (Megazyme, Bray, Ireland). Xylose was quantified by ion chromatography using a Dionex ICS-3000 system equipped with a CarboPac PA20 column (3 × 150 mm; Dionex Corporation, Sunnyvale, CA, USA). Ethanol was measured using the Megazyme K-ETOH assay kit according to the manufacturer’s instructions. Organic acids were quantified using a Dionex ICS-3000 ion chromatography system.

Ethanol yield on consumed sugar was calculated as:

$$Y_{EtOH∕S}=\frac{C_{EtOH,f}-C_{EtOH,0}}{C_{S,0}-C_{S,f}}$$

where *C*_EtOH_ and *C*_S_ represent the ethanol and fermentable-sugar concentrations, respectively, and subscripts 0 and *f* denote the initial and final values. For mixed-sugar cultures, the decreases in glucose and xylose concentrations were summed on a mass basis to obtain *C*_S,0_ − *C*_S,f_. For the coculture experiment, ethanol yield based on cellulose added was calculated as:

$$Y_{EtOH/cellulose}=\frac{C_{EtOH,f}}{C_{cellulose,0}}$$

where *C*_EtOH,f_ represents the final ethanol concentration and *C*_cellulose,0_ represents the initial cellulose concentration. The corresponding mass-based conversion ratios were calculated as:

CR(%)=Y×100

where *Y* represents the corresponding ethanol yield. All concentrations were expressed in g/L.

### 2.5. Statistical Analysis and Data Presentation

Bottle experiments were conducted using three independently inoculated biological replicates, and data are presented as the mean ± standard deviation (SD). No inferential statistical tests were performed; therefore, no significance threshold was applied. The coculture bioreactor experiment was conducted once and is presented as a preliminary fermentation time course without error bars.

## 3. Results and Discussion

### 3.1. Sugar Utilization by Seven Thermophilic Ethanologenic Strains

The capability to convert both hexoses and pentoses into ethanol is an important characteristic of an ethanologenic partner for CBP. We previously showed that the cellulosome of *A. thermocellus* retained greater cellulose-degrading activity than a commercial fungal cellulase under the tested concentrations of organic acids and ethanol [27]. Therefore, we sought to develop a coculture system consisting of a cellulose-degrading strain and a thermophilic ethanologenic partner. The seven thermophilic ethanologenic strains were evaluated for ethanol conversion from glucose and xylose. With either glucose or xylose as the sole carbon source, all seven strains grew and consumed sugar. *Thermoanaerobacter* sp. X514 showed the slowest growth and sugar consumption, whereas *T. italicus* DSM 9252 showed the fastest growth and sugar consumption under the tested conditions (Figure 1A,B,E,F).

*T. thermocopriae* DSM 11426, *T. brockii* subsp. *finnii* DSM 3389, and *T. xylanolyticum* DSM 7097 each showed sugar-to-ethanol conversion ratios above 32% with glucose (Figure 1C,D). The same three strains showed conversion ratios above 42% with xylose (Figure 1G,H). These values approached the theoretical mass conversion of approximately 51.1% for fermentation of glucose or xylose to ethanol. The results supported further evaluation of these strains as potential ethanologenic partners.

Carbon catabolite repression has been observed in some thermophilic ethanologenic strains under mixed-sugar conditions, although its occurrence depends on the organism and cultivation conditions [34]. The seven strains were therefore cultivated in a mixture containing 10 g/L glucose and 10 g/L xylose (Figure 2A–G). Both sugars were consumed during the early growth phase. During the later stage of fermentation, glucose was generally depleted more rapidly than xylose, except in the culture of *C. polysaccharolyticus* DSM 13641.

Sugar-to-ethanol conversion ratios above 32% were obtained for *T. brockii* subsp. *finnii* DSM 3389 at 35.7%, *T. xylanolyticum* DSM 7097 at 33.9%, *T. italicus* DSM 9252 at 32.5%, and *T. thermocopriae* DSM 11426 at 32.3% (Figure 2H). The conversion ratios were lower than the 47–49% reported for the engineered *T. italicus* Pentocrobe 411 under a different continuous-fermentation configuration [35]. These four strains were selected for the subsequent tolerance experiments. Under the conditions used here, X514 showed a lower conversion ratio than the four selected strains (Figure 1D and Figure 2H). Ethanol production by X514 is known to depend on cultivation conditions and medium composition [28,36]. Biochemical characterization of X514 identified AdhE and AdhB as abundant alcohol dehydrogenases during growth on glucose, with different cofactor specificities linking ethanol formation to cellular redox metabolism [37]. Accordingly, X514 was not included in the subsequent tolerance assays.

### 3.2. Effects of Pretreatment-Derived Inhibitors on Ethanol Production

Furfural and phenolic compounds generated during lignocellulose pretreatment can inhibit microbial growth and fermentation when present at sufficiently high concentrations [21,22,23]. In a coculture system, tolerance of the ethanologenic partner to these inhibitors may help maintain ethanol production when pretreated lignocellulosic material is used. The four selected strains were therefore cultivated in the presence of furfural (Figure 3) or *p*-hydroxybenzoic acid (Figure 4).

*T. brockii* subsp. *finnii* DSM 3389 (Figure 3A,B), *T. xylanolyticum* DSM 7097 (Figure 3C,D), and *T. thermocopriae* DSM 11426 (Figure 3G,H) retained growth and ethanol production in the presence of 20 mM furfural. *T. italicus* DSM 9252 showed a greater reduction under the same conditions (Figure 3E,F). DSM 3389 also retained final ethanol production in the presence of 20 mM *p*-hydroxybenzoic acid (Figure 4A,B), whereas greater reductions were observed for DSM 7097 (Figure 4C,D), DSM 9252 (Figure 4E,F), and DSM 11426 (Figure 4G,H). At 20 mM furfural or *p*-hydroxybenzoic acid, DSM 3389 showed higher mean ethanol production than the corresponding inhibitor-free culture (Figure 3B and Figure 4B). This non-monotonic response may reflect a concentration-dependent physiological response to inhibitor exposure. In the related thermophilic ethanologen *Thermoanaerobacter pseudethanolicus* 39E, furfural detoxification involves NADPH-dependent reduction of furfural to furfuryl alcohol [38], which may alter cofactor availability and ethanol formation under furfural stress.

In the presence of 30 mM furfural or 30 mM *p*-hydroxybenzoic acid, DSM 3389 retained approximately 80% of the ethanol production measured in the corresponding inhibitor-free control (Figure 3B and Figure 4B). The other strains showed lower ethanol production and stronger growth inhibition at these concentrations (Figure 3D,F,H and Figure 4D,F,H). Among the four strains tested, DSM 3389 showed the strongest observed tolerance to the two inhibitors under the experimental conditions. The concentrations and combinations of inhibitors in lignocellulosic hydrolysates vary with feedstock and pretreatment conditions [21,23,39]. The cellulosome isolated from *A. thermocellus* JYT01 was previously reported to tolerate up to 5 mM furfural and 50 mM *p*-hydroxybenzoic acid [27]. Although the previous study evaluated isolated cellulosome activity rather than microbial growth, those results complement the present evaluation of inhibitor tolerance in the ethanologenic partner. The observed tolerance of DSM 3389 supports its potential use in fermentation systems containing pretreatment-derived inhibitors.

### 3.3. Ethanol Tolerance of the Selected Strains

Ethanol accumulation inhibits the growth and metabolism of many microorganisms and remains an important limitation in thermophilic ethanol fermentation [12,13,14]. The four selected strains were therefore cultivated in the presence of exogenous ethanol concentrations ranging from 0 to 3.0% (*v*/*v*) (Figure 5). DSM 3389 (Figure 5A) and DSM 7097 (Figure 5B) retained growth at ethanol concentrations below 2.0% (*v*/*v*). Their fitted doubling times were shorter in the presence of 1.0% (*v*/*v*) ethanol. The doubling time of DSM 3389 decreased from 8.2 to 5.1 h, whereas that of DSM 7097 decreased from 5.7 to 4.1 h. Comparable decreases were not observed for DSM 9252 (Figure 5C) or DSM 11426 (Figure 5D).

At 1.0% (*v*/*v*) exogenous ethanol, DSM 3389 and DSM 7097 showed shorter fitted doubling times than the corresponding ethanol-free cultures. This pattern may reflect a concentration-dependent physiological response to sub-inhibitory ethanol exposure, consistent with previous observations that low concentrations of exogenous ethanol can alter fermentation-product formation in *Thermoanaerobacter* [40]. The approximate maximum exogenous ethanol concentration permitting growth was 2.5% (*v*/*v*) for all four strains under the tested conditions. Improving ethanol tolerance may further enhance their suitability for thermophilic ethanol fermentation [41,42].

### 3.4. Effects of Formate, Acetate, and Lactate on Ethanol Production

Organic acids may be introduced through biomass pretreatment or produced during fermentation. Their accumulation can influence microbial growth, intracellular carbon distribution, and ethanol production [23,25,26]. Previous experiments showed that externally supplied acetate and lactate affected ethanol production by X514 and the activity of the *A. thermocellus* cellulosome [27,36]. Sodium formate (Figure S1), sodium acetate (Figure S2), and sodium lactate (Figure S3) were therefore tested separately with the four selected strains.

All four strains remained capable of growth in media containing up to 100 mM of the tested sodium salts. In the presence of 100 mM sodium formate, final ethanol production was 73.5%, 66.1%, 51.6%, and 52.6% of the corresponding untreated controls for DSM 3389 (Figure S1B), DSM 7097 (Figure S1D), DSM 9252 (Figure S1F), and DSM 11426 (Figure S1H), respectively. DSM 3389 therefore showed the greatest retention of ethanol production at the highest formate concentration tested.

Sodium acetate and sodium lactate caused smaller changes in final ethanol production at concentrations up to 100 mM (Figures S2 and S3). Because sodium salts were used, these assays primarily evaluated the effects of the corresponding organic-acid anions under the initial medium pH rather than the toxicity of the undissociated acids. Previous work showed that acetate could stimulate ethanol production by *T. pseudethanolicus* 39E and X514, whereas lactate generally inhibited fermentation by strain 39E depending on the substrate and concentration [36]. Low concentrations of formate, acetate, and lactate were also reported to increase the activity of the cellulosome isolated from JYT01 [27]. Under the tested conditions, sodium acetate and sodium lactate at concentrations up to 100 mM did not markedly reduce final ethanol production by the four selected strains.

### 3.5. Mixed-Microbe Fermentation for Ethanol Production

The objective of the screening experiments was to select a thermophilic ethanologenic partner for the cellulose-degrading strain *A. thermocellus* JYT01. The strains were compared for sugar utilization (Figure 1 and Figure 2), furfural and *p*-hydroxybenzoic acid tolerance (Figure 3 and Figure 4), ethanol tolerance (Figure 5), and ethanol production in the presence of formate, acetate, and lactate (Figures S1–S3). Based on these combined observations, *T. brockii* subsp. *finnii* DSM 3389 was selected for coculture fermentation.

In a single preliminary bioreactor experiment, DSM 3389 and JYT01 were cocultivated in 2 L GS-2 medium containing 40 g/L Avicel at 60 °C and pH 7.0. Soluble sugar concentrations remained very low throughout fermentation, showing that soluble-sugar accumulation was limited under the tested conditions (Figure 6). This observation is consistent with sugar release not substantially exceeding overall sugar consumption, but it does not distinguish sugar uptake by DSM 3389 from that by JYT01. At 72 h, the ethanol concentration was 10.86 g/L, corresponding to a mass-based cellulose-to-ethanol conversion ratio of 27.2% based on the initial Avicel concentration. The same JYT01 strain has previously been evaluated under conditions selected to support cellulosome activity [27]. However, differences in cultivation conditions and experimental configurations preclude using the previous result as a direct quantitative monoculture benchmark. In the absence of a parallel JYT01 monoculture under the present conditions, the contribution of DSM 3389 to ethanol production cannot be determined.

Previous studies have shown that *A. thermocellus*-based cocultures can improve the utilization of released carbohydrates and modify fermentation-product distributions under specific cultivation conditions [15,16,17]. More recent studies also support the use of complementary thermophilic microorganisms for cellulose and hemicellulose conversion [18,19]. In the present study, DSM 3389 was selected through comparative phenotypic screening and subsequently evaluated in a defined coculture configuration with JYT01. Because Avicel is composed predominantly of cellulose, this experiment did not directly evaluate the potential contribution of DSM 3389 to xylose utilization. Studies using lignocellulosic substrates have shown that hemicellulose composition, substrate accessibility, and the metabolic complementarity of coculture partners can influence substrate conversion and ethanol production [18,19].

In this run, lactate and acetate concentrations reached 3.84 and 2.23 g/L, respectively (Figure 6), indicating that fermented carbon was distributed to organic acids as well as ethanol. This product distribution is consistent with the roles of the ethanol, acetate, and lactate pathways in ATP generation and redox balance in *A. thermocellus* [42]. Organic-acid formation therefore represents a relevant target for further optimization. Genome-scale metabolic analysis and population monitoring may help characterize the interactions between DSM 3389 and JYT01 and guide further development of this coculture [43]. Overall, the experiment provides a preliminary basis for evaluating this specific strain pairing, while its quantitative contribution to ethanol production requires matched monoculture controls and independently replicated fermentations.

## 4. Conclusions

In this study, seven thermophilic ethanologenic strains were evaluated for glucose and xylose utilization, ethanol production, and tolerance to representative pretreatment-derived inhibitors and fermentation products. Among the strains tested, *Thermoanaerobacter brockii* subsp. *finnii* DSM 3389 showed the highest observed sugar-to-ethanol conversion ratio of 35.7% from a glucose and xylose mixture and retained growth and/or ethanol production under the tested furfural, *p*-hydroxybenzoic acid, ethanol, formate, acetate, and lactate conditions. In a single preliminary pH-controlled fermentation containing 40 g/L Avicel, cocultivation of DSM 3389 and *A. thermocellus* JYT01 resulted in 10.86 g/L ethanol after 72 h, corresponding to a mass-based cellulose-to-ethanol conversion ratio of 27.2%. Lactate and acetate concentrations reached 3.84 and 2.23 g/L, respectively. These findings support the selection of DSM 3389 as a candidate ethanologenic partner and provide a preliminary basis for further evaluation of this strain pairing.

## Figures and Tables

**Figure 1:**
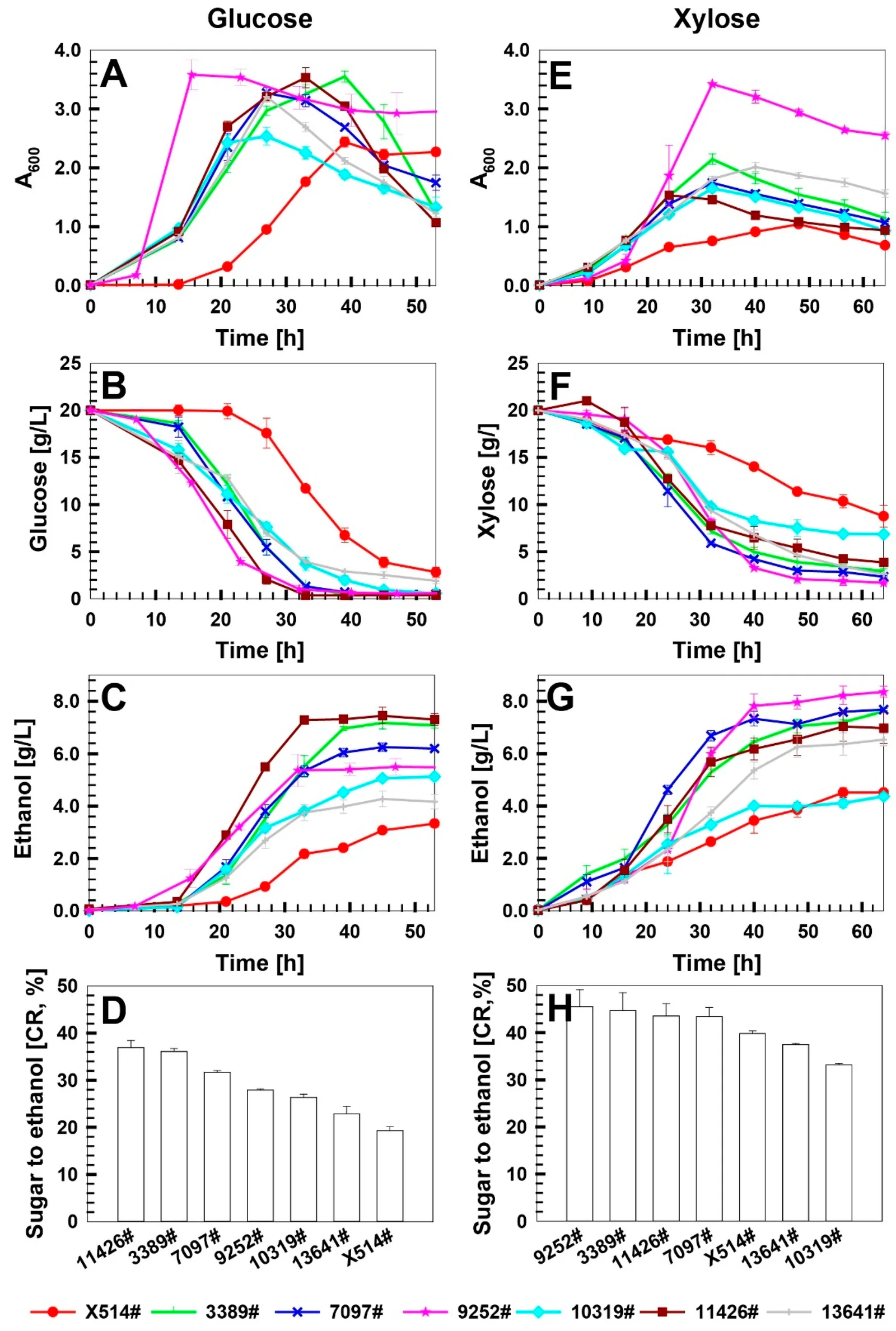
Sugar utilization by seven thermophilic ethanologenic strains. Seven thermophilic ethanologenic strains were cultivated in parallel in 30 mL GS-2 medium in 70 mL serum bottles supplemented with either 20 g/L glucose (**A**–**D**) or 20 g/L xylose (**E**–**H**) as the sole carbon source. Cell density (**A**,**E**), sugar concentration (**B**,**F**), and ethanol concentration (**C**,**G**) were measured every 8 h. The sugar-to-ethanol conversion ratios (CRs) are presented in (**D**,**H**). Values are means ± SD (n = 3 independent cultures); no inferential statistical tests were performed. The strains are indicated by DSM accession numbers or abbreviations.

**Figure 2:**
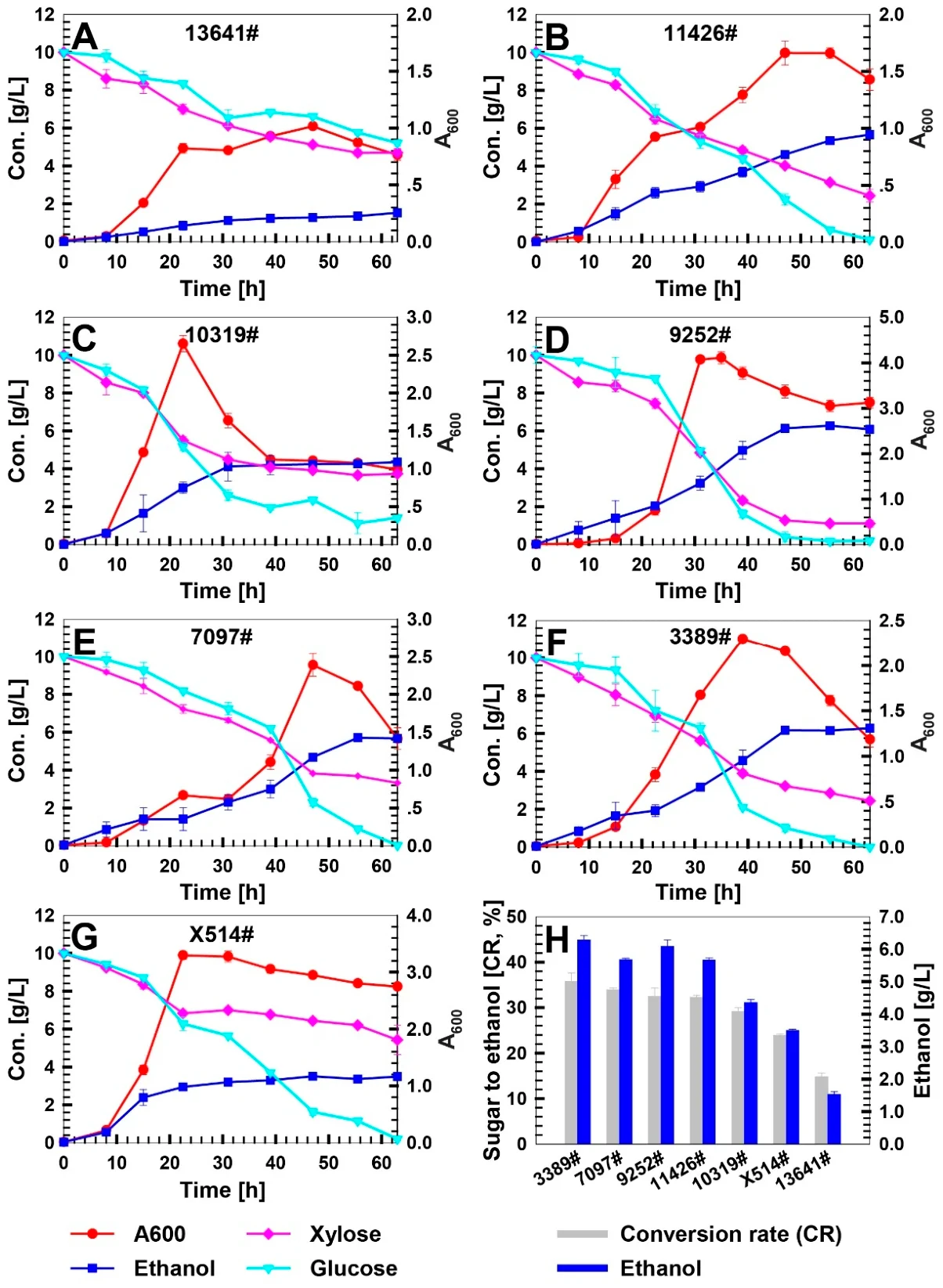
Glucose and xylose co-utilization by seven thermophilic ethanologenic strains. Seven thermophilic ethanologenic strains were cultivated in parallel in 30 mL GS-2 medium in 70 mL serum bottles supplemented with a mixture of glucose (10 g/L) and xylose (10 g/L). Cell density and sugar concentrations of *Caldanaerobius polysaccharolyticus* DSM 13641 (**A**), *Thermoanaerobacter thermocopriae* DSM 11426 (**B**), *Thermoanaerobacter wiegelii* DSM 10319 (**C**), *Thermoanaerobacter italicus* DSM 9252 (**D**), *Thermoanaerobacterium xylanolyticum* DSM 7097 (**E**), *Thermoanaerobacter brockii* subsp. *finnii* DSM 3389 (**F**), and *Thermoanaerobacter* sp. X514 (**G**) were measured every 8 h. Ethanol concentrations and sugar-to-ethanol conversion ratios (CRs) are shown in (**H**). Values are means ± SD (n = 3 independent cultures); no inferential statistical tests were performed.

**Figure 3:**
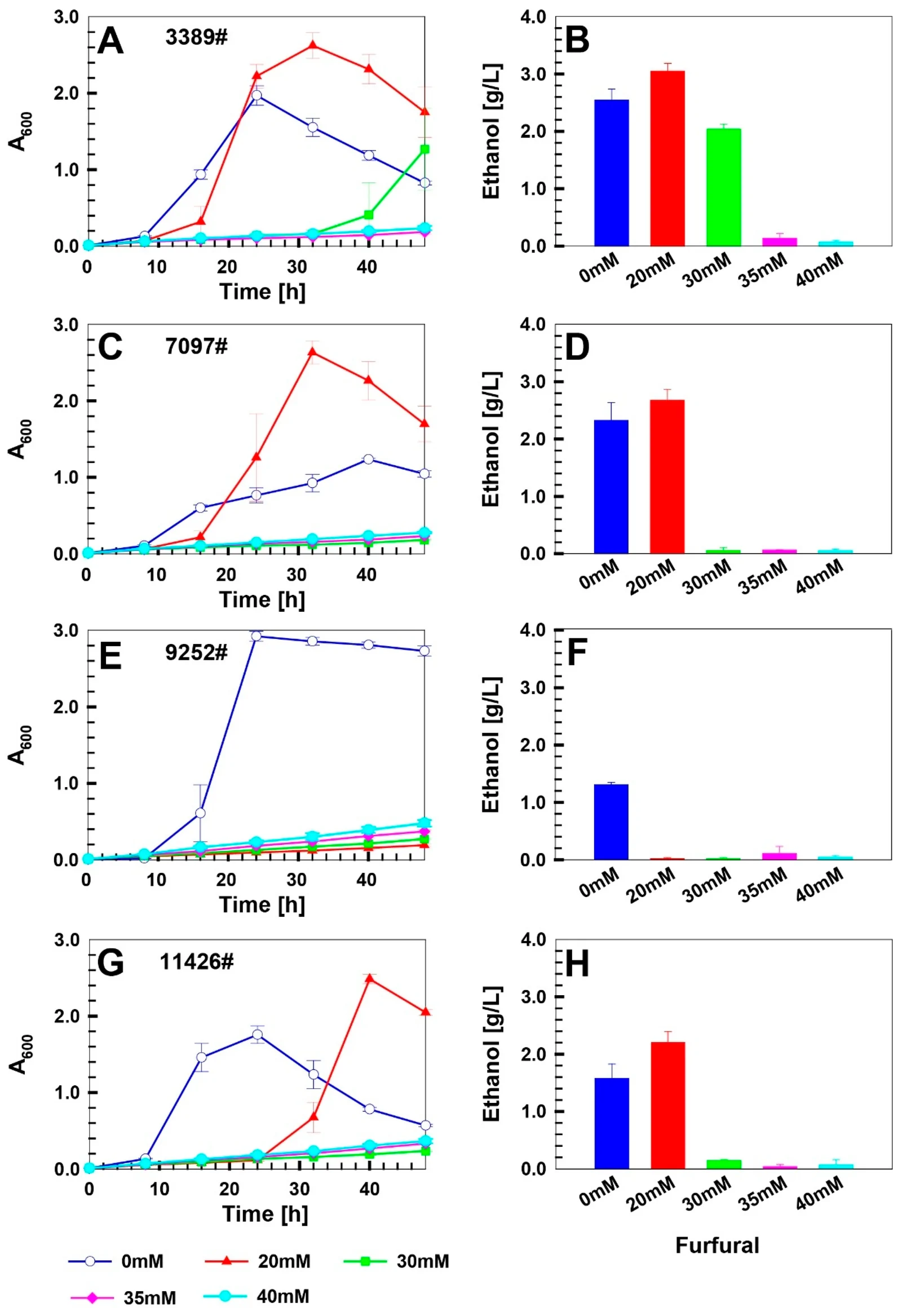
Effect of furfural on ethanol production by selected thermophilic ethanologenic strains. *T. brockii* subsp. *finnii* DSM 3389 (**A**,**B**), *T. xylanolyticum* DSM 7097 (**C**,**D**), *T. italicus* DSM 9252 (**E**,**F**), and *T. thermocopriae* DSM 11426 (**G**,**H**) were cultivated in 30 mL GS-2 medium in 70 mL serum bottles supplemented with 10 g/L glucose as the sole carbon source. Furfural was added to the medium at concentrations ranging from 0 to 40 mM. Cell density (**A**,**C**,**E**,**G**) and ethanol concentration (**B**,**D**,**F**,**H**) were measured every 8 h. Values are means ± SD (n = 3 independent cultures); no inferential statistical tests were performed.

**Figure 4:**
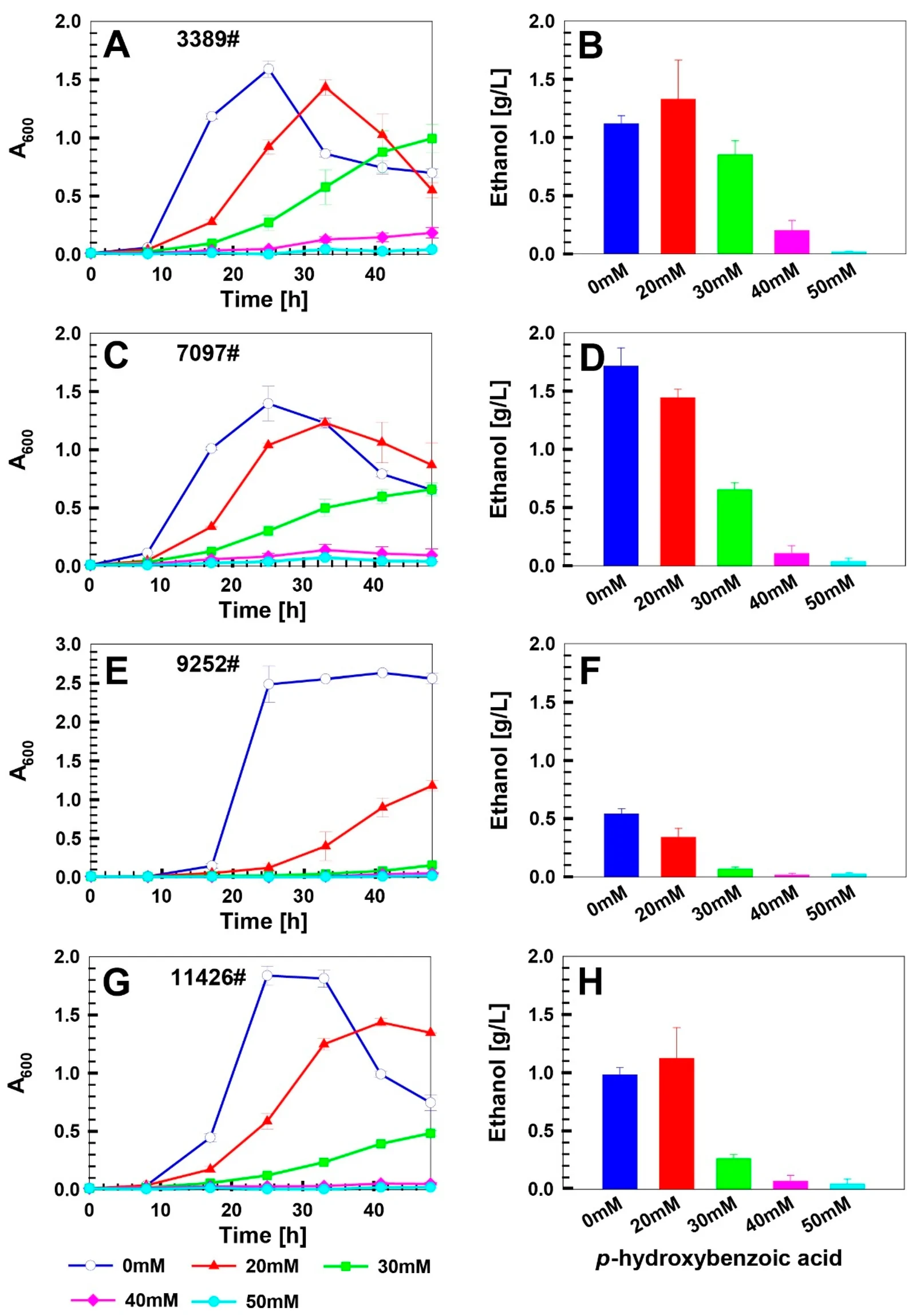
Effect of *p*-hydroxybenzoic acid on ethanol production by selected thermophilic ethanologenic strains. *T. brockii* subsp. *finnii* DSM 3389 (**A**,**B**), *T. xylanolyticum* DSM 7097 (**C**,**D**), *T. italicus* DSM 9252 (**E**,**F**), and *T. thermocopriae* DSM 11426 (**G**,**H**) were cultivated in 30 mL GS-2 medium in 70 mL serum bottles supplemented with 10 g/L glucose as the sole carbon source. *p*-Hydroxybenzoic acid was added to the medium at concentrations ranging from 0 to 50 mM. Cell density (**A**,**C**,**E**,**G**) and ethanol concentration (**B**,**D**,**F**,**H**) were measured every 8 h. Values are means ± SD (n = 3 independent cultures); no inferential statistical tests were performed.

**Figure 5:**
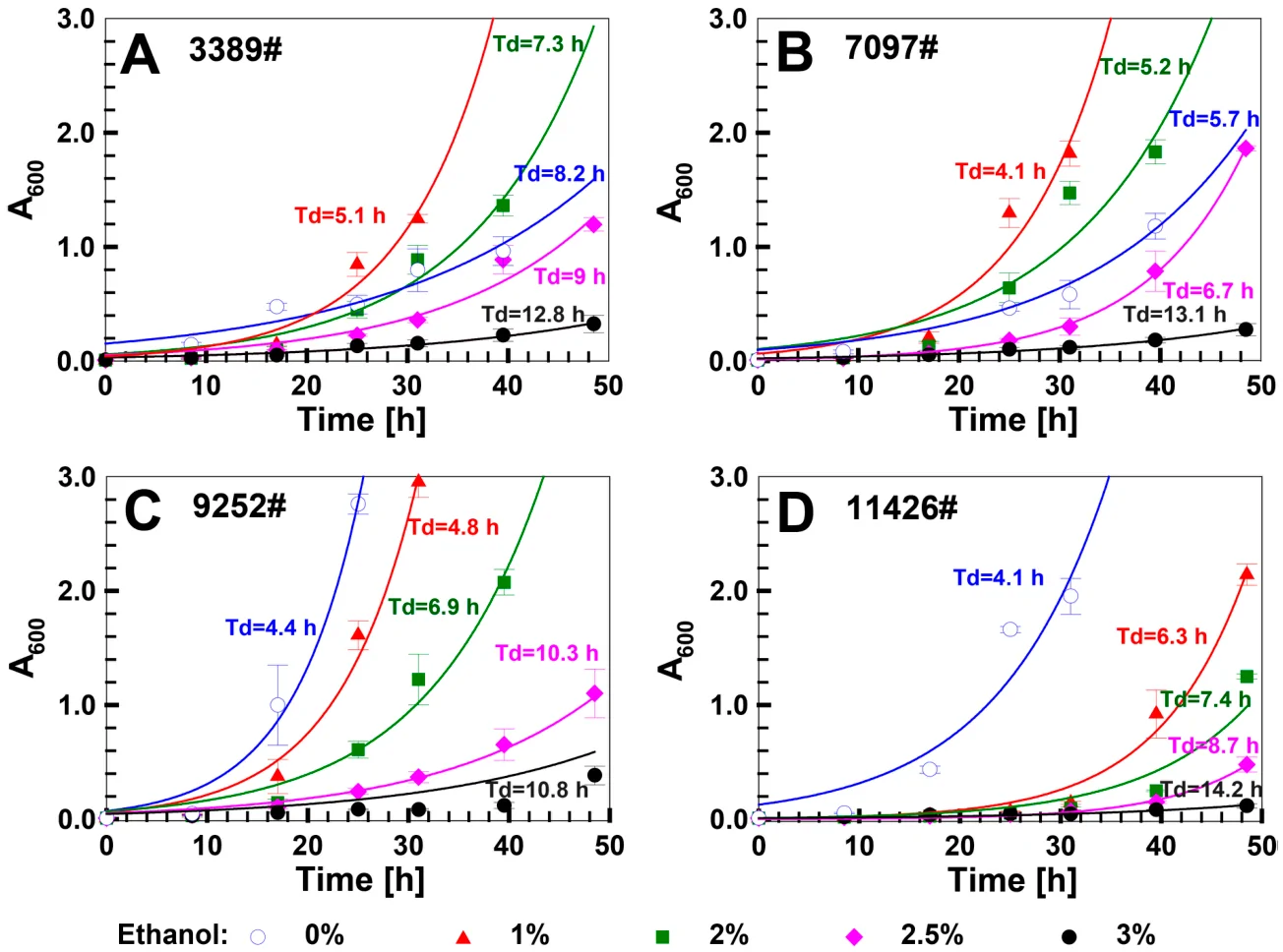
Ethanol tolerance of selected thermophilic ethanologenic strains. *T. brockii* subsp. *finnii* DSM 3389 (**A**), *T. xylanolyticum* DSM 7097 (**B**), *T. italicus* DSM 9252 (**C**), and *T. thermocopriae* DSM 11426 (**D**) were cultivated in 30 mL GS-2 medium in 70 mL serum bottles supplemented with 10 g/L glucose as the sole carbon source. Exogenous ethanol at concentrations ranging from 0 to 3% (*v*/*v*) was added to the medium. Growth curves were fitted using the exponential growth equation *Y = ae^bX^*. The doubling time (Td) of each strain was calculated as ln(2)/*b* and is indicated on each curve. Values are means ± SD (n = 3 independent cultures); no inferential statistical tests were performed.

**Figure 6:**
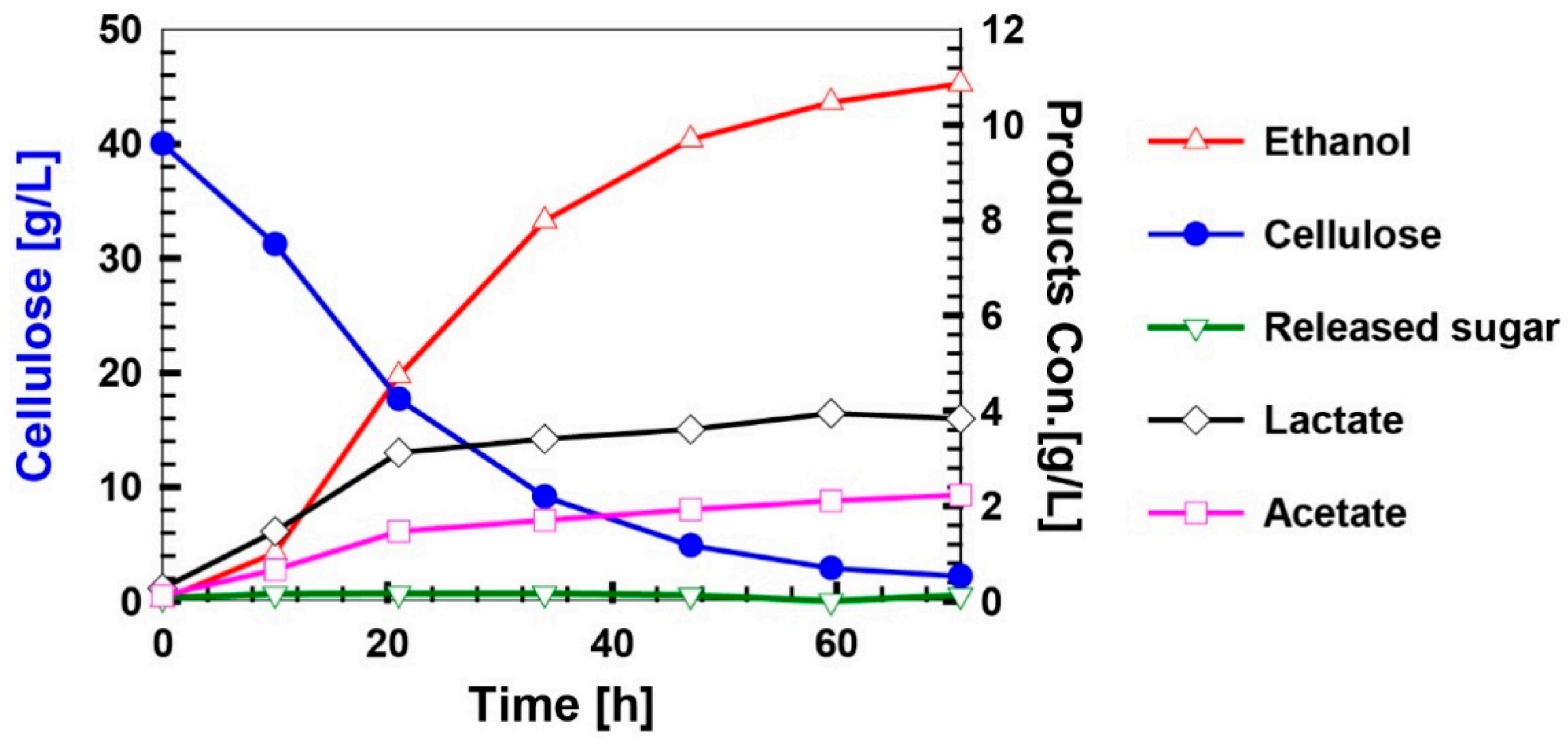
Coculture fermentation of *T. brockii* subsp. *finnii* DSM 3389 and *A. thermocellus* JYT01. *T. brockii* subsp. *finnii* DSM 3389 and *A. thermocellus* JYT01 were inoculated at an initial ratio of 1:1 (*v*/*v*) into a 5 L bioreactor with a 2 L working volume. Fermentation was performed at 60 °C with the pH maintained at 7.0. Cellulose, soluble sugar, ethanol, and organic acid concentrations were measured every 12 h. Data are from a single preliminary bioreactor run and are shown without error bars.

## Data Availability

The raw data supporting the conclusions of this article will be made available by the authors on request.

## Supplementary Materials

The following supporting information can be downloaded at: https://www.mdpi.com/article/10.3390/microorganisms14102164/s1, Figure S1: Effect of sodium formate on ethanol production by selected thermophilic ethanologenic strains; Figure S2: Effect of sodium acetate on ethanol production by selected thermophilic ethanologenic strains; Figure S3: Effect of sodium lactate on ethanol production by selected thermophilic ethanologenic strains.
