# Supplementary material for: Cellulosic Ethanol Production Through Cocultivation of Thermoanaerobacter brockii subsp. finnii DSM 3389 and Acetivibrio thermocellus JYT01
Source: Microorganisms. 2026 Sep 25;14(10):2164. doi: 10.3390/microorganisms14102164 (PMC13707418; doi:10.3390/microorganisms14102164)
Supplement: Supplementary file 1 [file microorganisms-14-02164-s001.zip › microorganisms-4575589-supplementary.pdf]

Cellulosic Ethanol Production through Cocultivation of *Thermoanaerobacter brockii* subsp. *finnii* DSM 3389 and *Acetivibrio thermocellus* JYT01

Zili Qiu[1,2], Chenggang Xu[2*], Zhenxing Ren[1*]

[1]Institute of Applied Chemistry, Shanxi University, Taiyuan 030006, Shanxi Province, China

[2]College of Animal Science and Technology & College of Veterinary Medicine of Zhejiang A&F University, Key Laboratory of Applied Technology on Green-Eco-Healthy Animal Husbandry of Zhejiang Province, Zhejiang Provincial Engineering Research Center for Animal Health Diagnostics & Advanced Technology, Zhejiang International Science and Technology Cooperation Base for Veterinary Medicine and Health Management, China-Australia Joint Laboratory for Animal Health Big Data Analytics, Hangzhou 311300, Zhejiang Province, China

* Correspondence: xucg@zafu.edu.cn (C.X.); zxren@sxu.edu.cn (Z.R.)

SUPPLEMENTARY MATERIAL

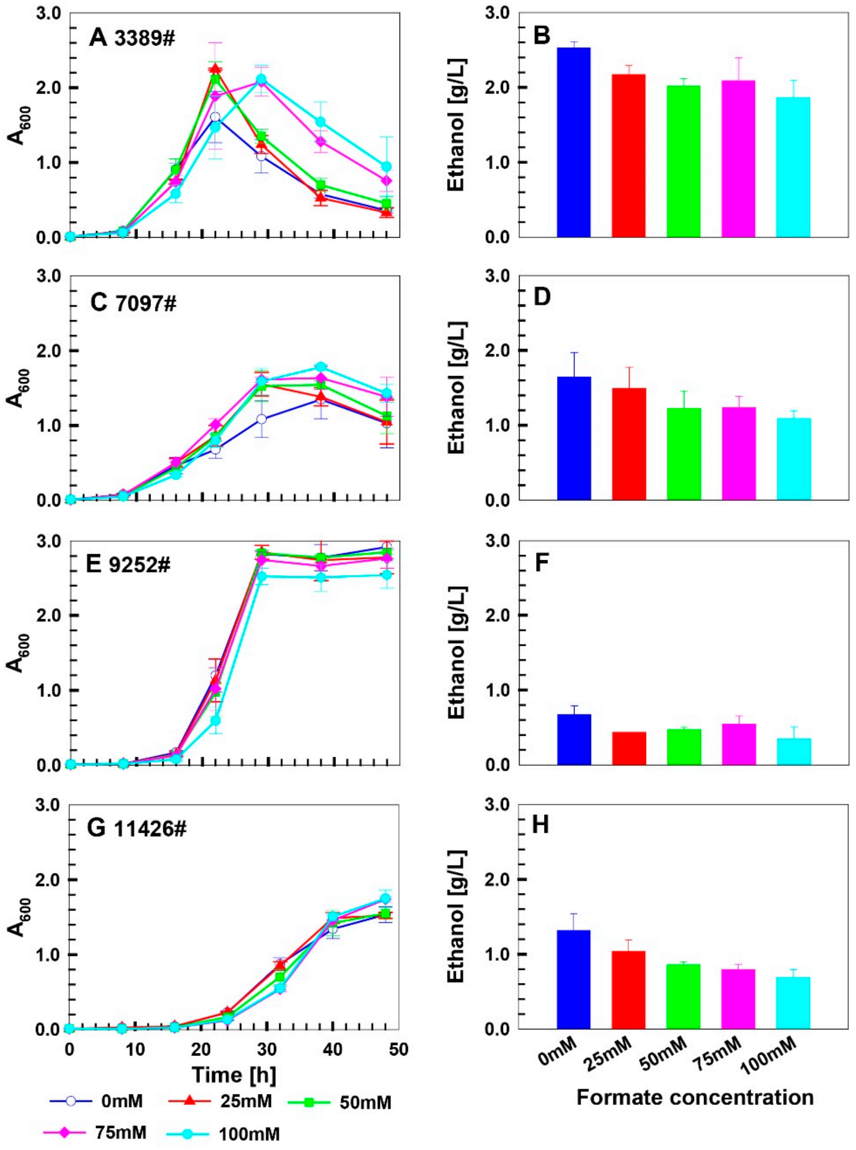

Figure S1. Effect of sodium formate on ethanol production by selected thermophilic ethanologenic strains. *T. brockii* subsp. *finnii* DSM 3389 (**A** and **B**), *T. xylanolyticum* DSM 7097 (**C** and **D**), *T. italicus* DSM 9252 (**E** and **F**), and *T. thermocopriae* DSM 11426 (**G** and **H**) were cultivated in 30 mL GS-2 medium in 70 mL serum bottles supplemented with 10 g/L glucose as the sole carbon source. Sodium formate was added to the medium at concentrations ranging from 0 to 100 mM. Cell density (**A**, **C**, **E**, and **G**) and ethanol concentration (**B**, **D**, **F**, and **H**) were measured every 8 h. Values are means ± SD (n = 3 independent cultures); no inferential statistical tests were performed.

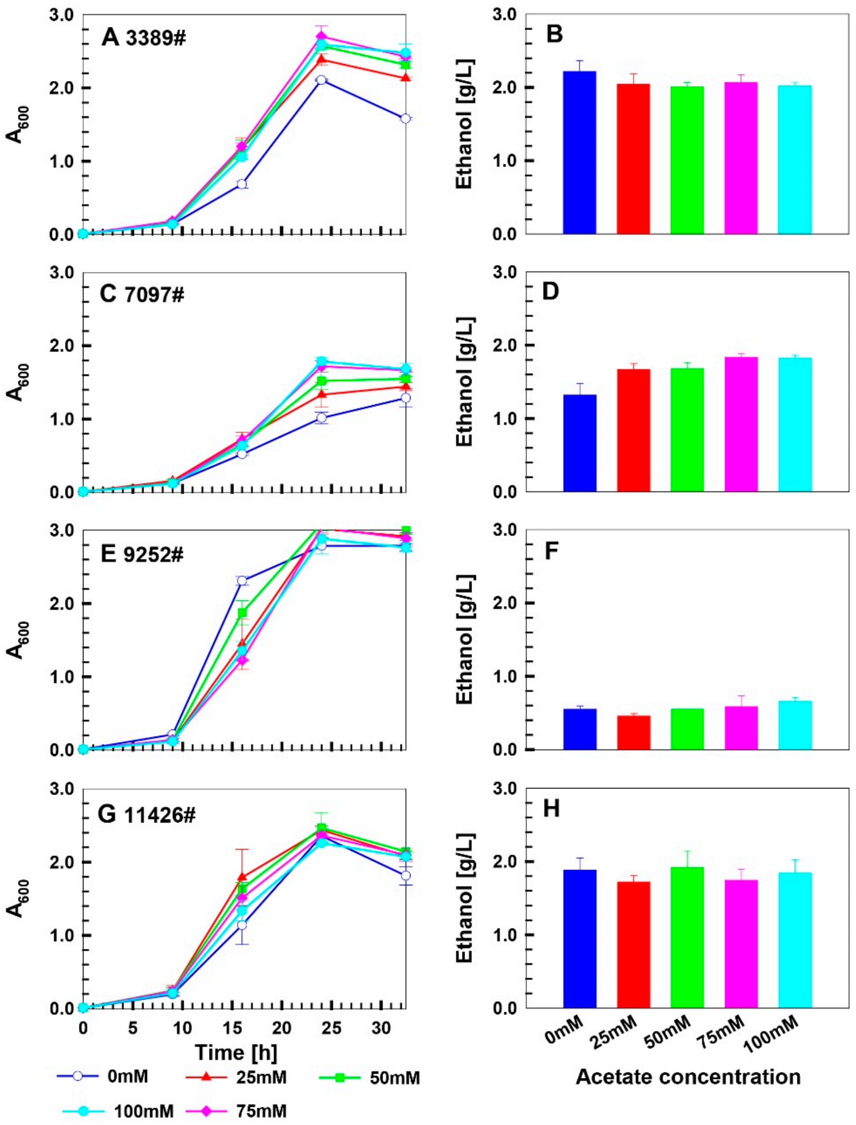

Figure S2. Effect of sodium acetate on ethanol production by selected thermophilic ethanologenic strains. *T. brockii* subsp. *finnii* DSM 3389 (**A** and **B**), *T. xylanolyticum* DSM 7097 (**C** and **D**), *T. italicus* DSM 9252 (**E** and **F**), and *T. thermocopriae* DSM 11426 (**G** and **H**) were cultivated in 30 mL GS-2 medium in 70 mL serum bottles supplemented with 10 g/L glucose as the sole carbon source. Sodium acetate was added to the medium at concentrations ranging from 0 to 100 mM. Cell density (**A**, **C**, **E**, and **G**) and ethanol concentration (**B**, **D**, **F**, and **H**) were measured every 8 h. Values are means ± SD (n = 3 independent cultures); no inferential statistical tests were performed.

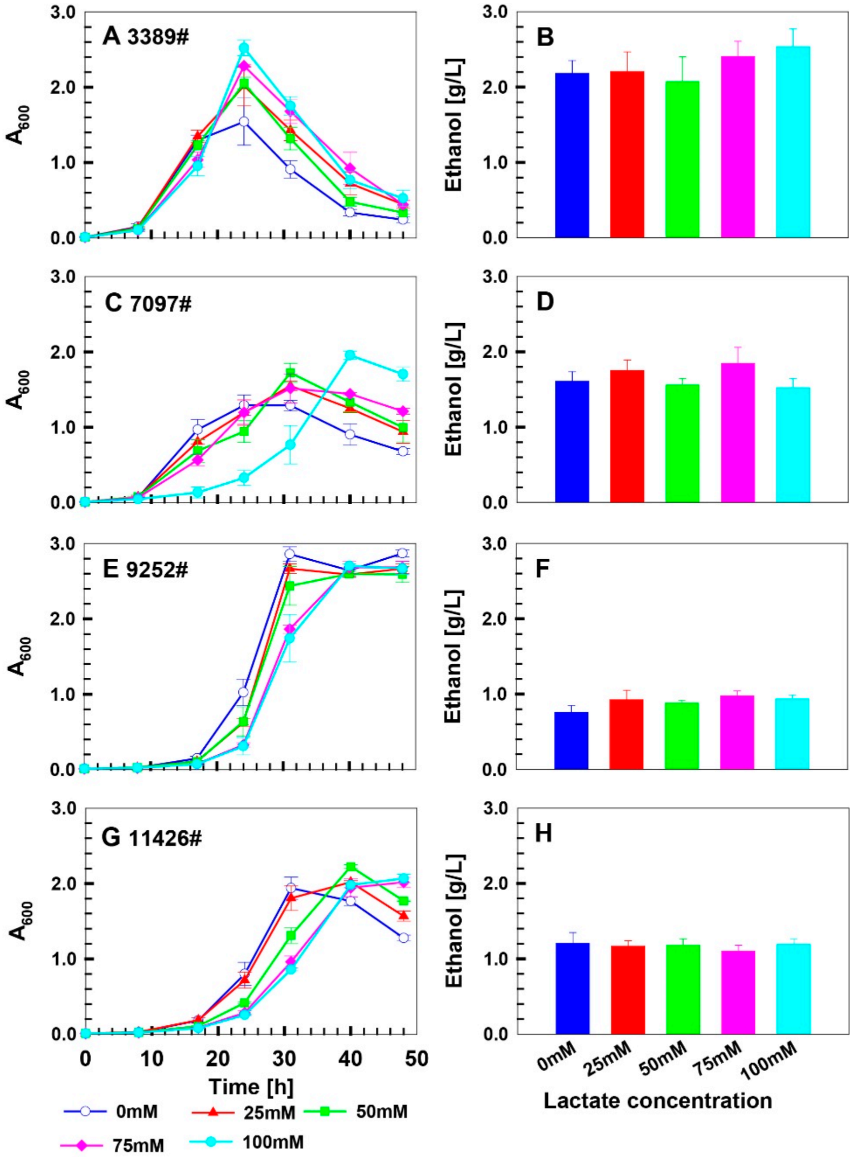

Figure S3. Effect of sodium lactate on ethanol production by selected thermophilic ethanologenic strains. *T. brockii* subsp. *finnii* DSM 3389 (**A** and **B**), *T. xylanolyticum* DSM 7097 (**C** and **D**), *T. italicus* DSM 9252 (**E** and **F**), and *T. thermocopriae* DSM 11426 (**G** and **H**) were cultivated in 30 mL GS-2 medium in 70 mL serum bottles supplemented with 10 g/L glucose as the sole carbon source. Sodium lactate was added to the medium at concentrations ranging from 0 to 100 mM. Cell density (**A**, **C**, **E**, and **G**) and ethanol concentration (**B**, **D**, **F**, and **H**) were measured every 8 h. Values are means ± SD (n = 3 independent cultures); no inferential statistical tests were performed.
